# Anticancer compound XL765 as PI3K/mTOR dual inhibitor: A structural insight into the inhibitory mechanism using computational approaches

**DOI:** 10.1371/journal.pone.0219180

**Published:** 2019-06-27

**Authors:** Mohd Rehan

**Affiliations:** 1 King Fahd Medical Research Center, King Abdulaziz University, Jeddah, Saudi Arabia; 2 Department of Medical Laboratory Technology, Faculty of Applied Medical Sciences, King Abdulaziz University, Jeddah, Saudi Arabia; University of Minnesota Twin Cities, UNITED STATES

## Abstract

The PI3K-AKT-mTOR pathway is often a commonly disrupted pathway in human cancer and, therefore, it is widely exploited for cancer therapy. The inhibitors for the important proteins of the pathway including PI3K and mTOR have been increasingly designed. The dual inhibitors targeting PI3K and mTOR both have proven to be more effective than those targeting single protein only. An orally-active compound XL765 is well established as PI3K/mTOR dual inhibitor and have shown *in vitro* and *in vivo* anticancer activity against a variety of cancer types and is undergoing clinical trials. The present study explored the exact binding pose and the the interactive forces holding XL765 within the active sites of PI3Kγ and mTOR using molecular docking analyses. The XL765 interacting residues of both the proteins were delineated and the degree of participation in binding was estimated by various methods. In the process, among the interacting residues of PI3Kγ, the Lys-890 and the Met-953 were recognized as the key residues involved in XL765 binding. While, in mTOR case, the Trp-2239 was recognized as the key residue playing role in the XL765 binding. In order to explore the better inhibitors, the study also generated combinatorial chemical library by modifying the scaffold considered from XL765. The virtual screening of the generated compound library led to identification of six novel promising compounds proposed as PI3K/mTOR dual inhibitors. Thus, the present work will through light on the drug inhibitory mechanism of XL765 for PI3K and mTOR, and will also assist in designing novel efficacious drug candidates.

## Introduction

Cancer is world-wide deadly disease, and in 2012 alone, it is bringing about 14.1 million new cancer occurrences and 8.2 million deaths. It is expected that these figures will rise to whopping 22 million new cancer incidences and 13 million deaths within the succeeding two decades [1]. Considering the global devastation the disease is causing, the demand for the novel and effective drugs are far from complete. The recent advancement in cancer research led to augmented understanding including the roles various molecular pathway play explicitly in onset and progression of the disease. The PI3K-AKT-mTOR pathway is an important growth signaling pathway and its constant activation in various cancer types has qualified it for serving as fascinating target for anti-cancer therapy [2–5]. Numerous attempts have been increasingly made in recent years for designing novel inhibitors against key signaling proteins in the pathway including PI3K, AKT, and mTOR [6–11].

The Phosphatidylinositol-4,5-bisphosphate 3-kinase (PI3Ks) are members of family of lipid and serine/threonine kinases, which phosphorylate 3' OH group of Phosphatidylinositol-4,5-bisphosphate (PIP2) to generate Phosphatidylinositol-3,4,5-trisphosphate (PIP3). The PI3Ks regulate cellular metabolism and growth by phosphorylating downstream effectors and adaptors through the second messenger PIP3 [12]. However, the constant activation and/or over-expression of PI3K turns into disrupted cellular functions which lead to cancerous conditions [3, 13–17]. PI3Ks are divided into three groups viz., I, II, and III based on their sequences and substrate specificity [3, 18–19]. The class I PI3Ks are the most implicated in cancer [20] and are further grouped into two categories Class IA and IB based on their regulatory subunits and the activation mechanism. Class IA PI3Ks contain a regulatory and a catalytic subunits, namely p85 and p110, respectively. The class IA p110 subunit is found in four isoforms α, β, γ and δ. All p110 catalytic subunits share a common basic structure with a C2 domain, a helical domain, and a catalytic domain [21]. All class IA p110 subunits are activated by receptor tyrosine kinases (RTKs) [22, 2], except p110γ which is unique in being activated alone by G-proteins coupled receptors (GPCRs) [23]. The constant activation of class 1A PI3Ks due to mutation are often detected in various human cancers [3, 13–17].

The mTOR, a cytosolic serine-threonine kinase, is a member of PI3K-related kinase family (PIKKs). The mTOR stands for mammalian target of rapamycin and was so named as it was first discovered as a mammalian homolog to a yeast protein called TOR, an acronym for “target of rapamycin” [24]. There are two related proteins in yeast called TOR1 and TOR2, whereas mammalian cells have one protein mTOR which uses varying input proteins to form two multi-protein complexes, mTOR complex 1 (mTORC1) and mTOR complex 2 (mTORC2) carrying out different signaling effects [25–26]. The kinase domain of mTOR (approx. 550 residues) consist of N-terminal lobe (N lobe) and a larger C-terminal lobe (C lobe) and the ATP binding site or catalytic site in the cleft between them [27–28]. The mTOR controls metabolism and growth of the cells, and thus, is a suitable drug target in variety of cancer types [29–30]. The rapamycin, the first known inhibitor of mTOR, associates with its intracellular receptor FKBP12 and inhibits mTOR allosterically [31–32]. The FKBP12-rapamycin complex directly interacts with FKBP12-rapamycin binding (FRB) domain to inhibit mTOR function [32]. The rapamycin binds to only one mTOR complex, mTORC1 and inhibit mTORC1 related signaling functions only [33]. This led to the discovery of ATP-competitive mTOR inhibitors [34] which bind to both mTORC1and mTORC2 and thus, inhibit kinase-dependent function of mTOR [35–38]. The inhibitors targeting either PI3K or mTOR alone has few shortcomings. The inhibitors targeting only PI3K does not abrogate mTOR functions and also the specific mTOR inhibition prompt PI3K signaling through a negative feedback mechanism [39–41]. To overcome these limitations, various dual PI3K/mTOR inhibitors are discovered which concomitantly inhibit both the proteins [38] and some of these dual inhibitors have entered clinical trials [42–43].

**Fig 1:**
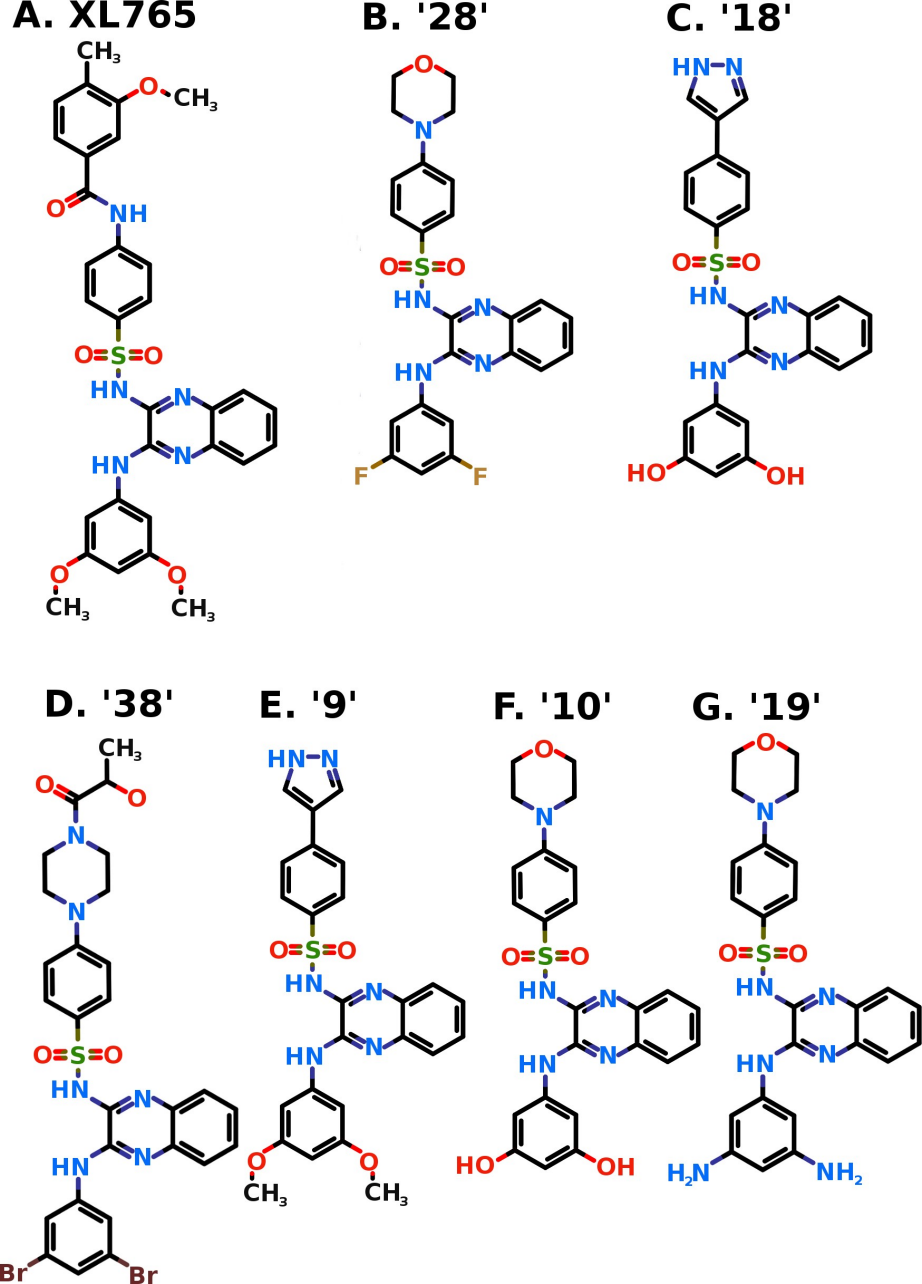
Two dimensional structure of XL765 (Panel A) and generated promising compounds 28 (Panel B), 18 (Panel C), 38 (Panel D), 9 (Panel E), 10 (Panel F), and 19 (Panel G). The nitrogen (N), oxygen (O), Fluorine (F), and Sulfur (S) atoms are shown in blue, red, tan, and khaki colors respectively.

XL765 (Exelixis, Inc., South San Francisco, CA USA) (Fig 1A) is an orally-active, potent and selective class-I PI3K/mTOR inhibitor which has demonstrated broad anti-cancer efficacy [44]. The IC_50_ value of XL765 for PI3K p110γ isoform is 9 nM (minimum of those for all p110 isoforms α, β, γ, and δ) and for mTOR is 157 nM [45]. The XL765 shows antitumor activity alone or in combination with temozolomide in variety of diverse xenografts and animal models [46–49]. This drug is undergoing clinical trials alone and in combination with other drugs for variety of cancer types [44, 50]. A few recent studies involving multiple inhibitors along with XL765 includes mTOR consensus docking [51] and PI3K isoform specific docking [52] were performed and conclusions were drawn from consensus docking poses of multiple inhibitors. However, in the present work, the attempts are made to explore the exact binding pose, interacting residues, molecular interactions, and the key interacting residues of PI3Kγ and mTOR using XL765 docking. Further, in order to explore the better inhibitors, study also generated a combinatorial chemical library by modification of scaffold considered from XL765 followed by its virtual screening against PI3Kγ and mTOR.

## Materials and methods

### Data retrieval

The three dimensional coordinates of XL765 was obtained from PubChem compound database (CID: 49867926), while the structures of proteins were retrieved from Protein Data Bank (PDB): PI3Kγ p110 with PDB Id, 3L54 and mTOR with PDB Id, 4JT6 respectively. The kinase domain of mTOR (residues ranging from 1867 to 2436) was considered in the study and used for all analyses. Both of the retrieved structures were co-complex structures with bound ligands (PI3Kγ with bound LXX, mTOR with bound PI-103), and these bound ligands were used as clues for catalytic site grid generation in molecular docking.

### Molecular docking and modification of chemical compound

All the molecular docking simulations were performed by Dock v.6.5 [53]. The pre-processing of proteins and ligands, called structure preparation, required as input for docking was performed by Chimera v.1.6.2 [54]. The chemical compounds were modified using MarvinSketch v.18.4, ChemAxon (http://www.chemaxon.com/products/marvin).

### Analyses of docked protein-ligand complex

The docked complexes of protein and ligand were visually analyzed by PyMOL v.1.3 [55] and illustrations were prepared. The molecular interactions between the proteins and ligands were analyzed and illustrated by Ligplot+ v.1.4.3 program [56–57]. For a residue, the degree of taking part in binding was evaluated by loss in accessible surface area (ASA). A residue is said to be taking part in ligand binding if it loses more than 10 Å^2^ ASA due to binding [58]. All the ASA calculations of the protein-ligand complexes and the unbound proteins were performed by Naccess v.2.1.1 [59]. To check the binding strength of the proteins towards the ligand, the binding energies and dissociation constants were calculated by X-Score v.1.2.11 [60–61].

### Drug-likeness and pharmacokinetic predictions

The “pkCSM-pharmacokinetics” online web-server (http://biosig.unimelb.edu.au/pkcsm/) was used for predictions of drug-likeness and pharmacokinetic properties absorption, distribution, metabolism, excretion, and toxicity [62]. This method uses graph based signature for a chemical compound containing all sets of distance patterns between atoms. These signatures of compounds were used to predict regression and classification models for multiple pharmacokinetic properties.

### Enrichment evaluation for molecular docking

Enrichment procedure is used for molecular docking evaluation and it measures how active compounds rank versus a background of decoys. Decoys act as negative controls and should not actually bind. Directory of useful decoys (DUD, http://dude.docking.org), Shoichet Laboratory in the Department of Pharmaceutical Chemistry at the University of California, San Francisco (UCSF), USA was used for generating decoys for the proposed inhibitors [63]. The DUD decoys are similar to known ligands physically but dissimilar topologically. DUD uses 2-D similarity fingerprints to minimize the topological similarity between decoys and ligands.

The enrichment factor (EF) is defined as the ratio between the percentage of active compounds in the selected upper subset and the percentage in the entire database [64].

$$EF=\frac{Hits(sample)/N(sample)}{Hits(database)/N(database)}$$

where Hits(sample) is the number of target-specific active compounds picked by docking in sample dataset (at a specific % level of ranked database, let’s say 20%); N(sample) is the total number of compounds in sample dataset (upper 20% of the ranked database); Hits(database) is the total number of target-specific active compounds in the database; N(database) is the total number of compounds in the database.

## Results and discussion

### Molecular docking study of XL765 with PI3Kγ

**Fig 2:**
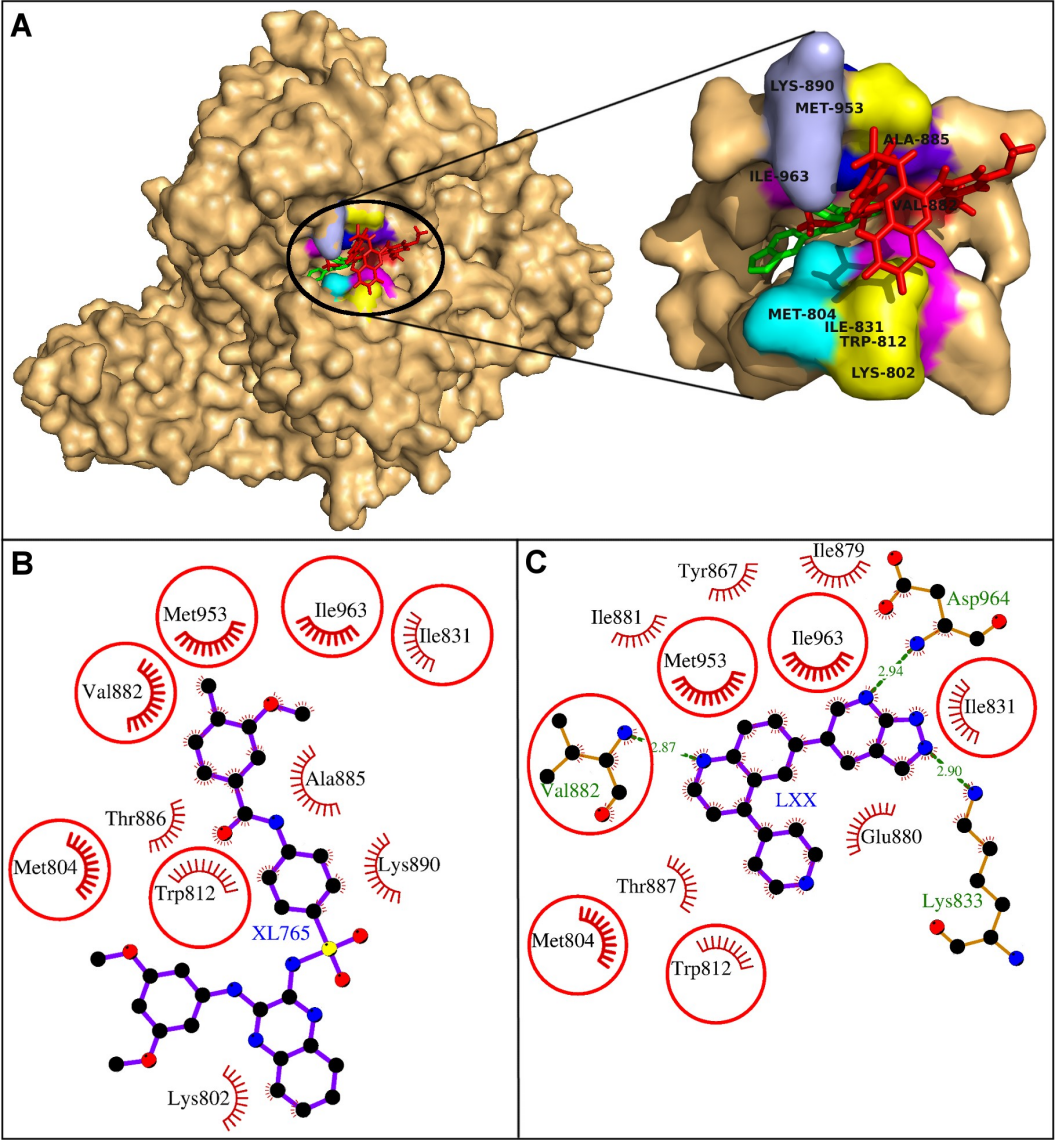
Human PI3Kγ with docked XL765. *A*. Human PI3Kγ and XL765 are depicted in surface and red sticks representations. The binding site is blown-up and the interacting residues are labeled and shown as surface in different colors. *B-C*: Comparative binding analysis of XL765 and the native ligand, LXX. The hydrogen bonds are presented as green-dashed lines labeled with bond lengths and the residues exerting non-bonding interactions are shown as red arcs. The interacting residues common for both the ligands are encircled.

The molecular docking study showed that the XL765 binding pocket was lined by the residues Lys-802, Met-804, Trp-812, Ile-831, Val-882, Ala-885, Thr-886, Lys-890, Met-953, and Ile-963. (Fig 2, Table 1). These 10 residues exerted 28 non-bonding interactions on the drug and stabilized the drug-protein complex (Table 1). The high absolute values of dock score, binding energy, and dissociation constant showed good quality binding (Table 2). The residue Lys-890 showed maximum ΔASA (59.19 Å^2^) due to binding which meant it had great involvement in drug binding (Table 1). Another important residue Met-953 was involved in maximum of eight non-bonding interactions with the drug suggesting its critical role in stabilizing the the drug-protein complex (Table 1). These residues Lys-890 and Met-953 were identified as the key residues required for XL765 binding in the present study.

**Table 1:** The PI3Kγ residues interacting with XL765 are listed with the number of non-bonding interactions and ΔASA.

Interacting residues	Non-bonding interactions	ΔASA (Å^2^)
Lys-802	3	48.45
Met-804	1	25.32
Trp-812	5	49.81
Ile-831	1	22.3
Val-882	2	14.1
Ala-885	2	26.79
Thr-886	1	22.98
Lys-890	4	59.19
Met-953	8	35.83
Ile-963	1	20.35

**Table 2:** The binding strengths of XL765, and compounds 28, 18, 38, 9, 10, 19, and respective native ligand with two cancer signaling proteins are presented with the number of molecular interactions and other scores. The number of residues participating in non bonding interactions are shown in parentheses. The 'K_d_' denotes the dissociation constant.

	Compounds	Rank of compound	Hydrogen bonds	Non-bonding interactions	Dock Score	Binding energy (KCal/Mol)	pK_d_
PI3K	XL765			28(10)	-35.16	-7.89	5.79
	28	1	2	37(13)	-48.59	-9.14	6.70
	18	5	1	45(13)	-45.12	-8.49	6.23
	38	6	4	37(13)	-42.95	-9.98	7.32
	9	14		38(10)	-39.45	-7.82	5.73
	10	20	2	52(11)	-34.44	-8.49	6.23
	19	16	2	24(8)	-38.32	-7.45	5.46
	Native		3 (3)	51 (13)		−10.28	7.54
mTOR	XL765		1	37(11)	-41.17	-8.56	6.28
	28	5		31(11)	-48.32	-9.16	6.71
	18	7	2	39(15)	-46.96	-8.80	6.45
	38	12		31(10)	-44.91	-8.82	6.47
	9	9		30(10)	-46.26	-8.23	6.03
	10	11	2	26(11)	-45.91	-8.71	6.39
	19	16	1	42(13)	-43.70	-9.46	6.94
	Native		3 (3)	46(11)		−8.88	6.51

On comparing the binding of the docked XL765 with that of native ligand LXX, six residues Met-804, Trp-812, Ile-831, Val-882, Met-953, and Ile-963 were found common for both the ligands (Fig 2B and 2C). Of the common residues, Met-953 was showing maximum non-bonding interactions. The Val-882 formed hydrogen bond with the bound ligand LXX, however, in the present work, it was participating in non-bonding interactions and showed adequate ΔASA (14.1 Å^2^) due to binding. The rest four residues showed their importance in binding for their involvement in non-bonding interactions and ample ΔASA due to binding. From these findings, the relevance of the common residues became obvious and this also showed that XL765 was also engaging the residues common to that of the native ligand and thus inhibiting the protein [44–45].

### Molecular docking study of XL765 with mTOR

**Fig 3:**
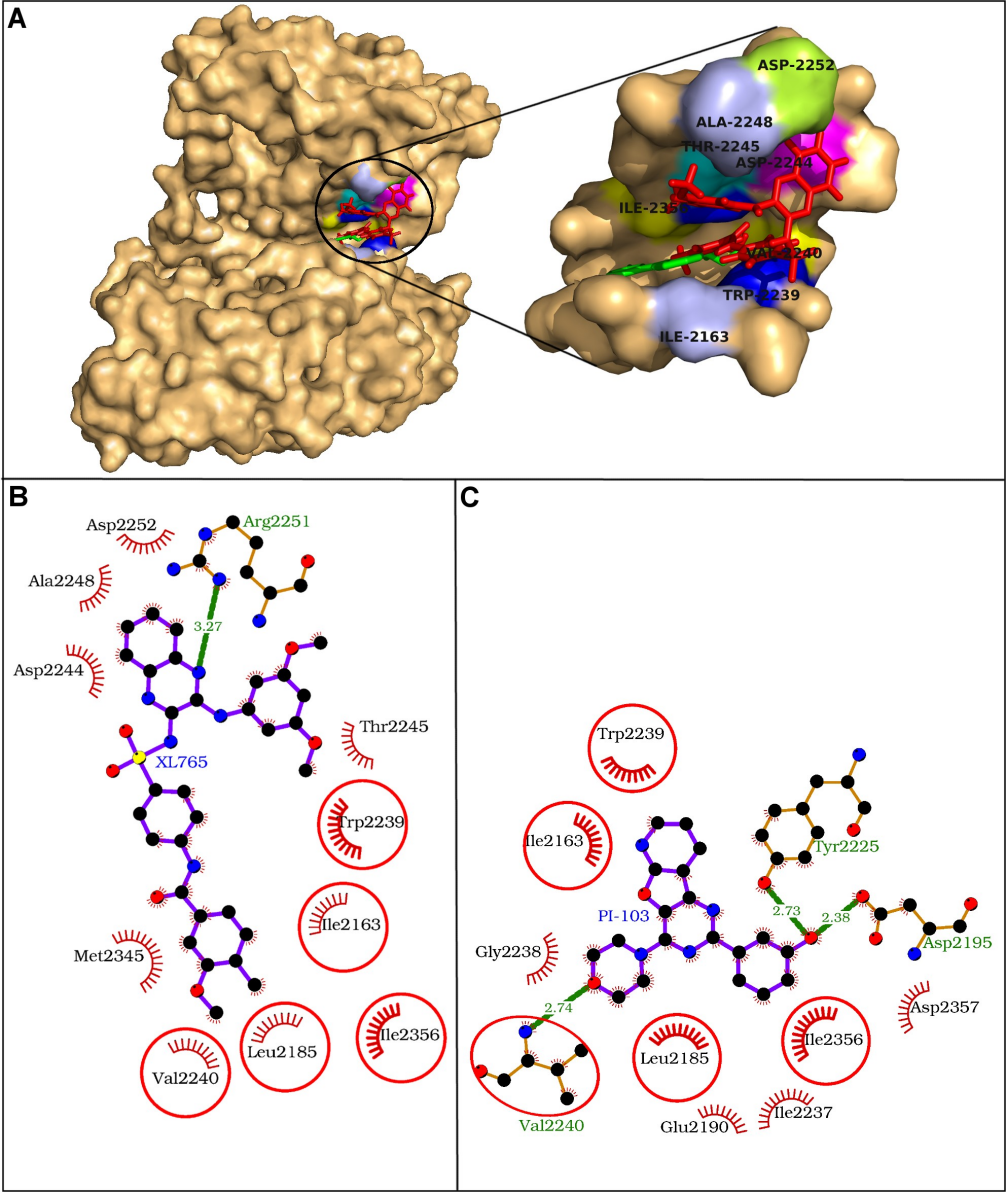
Human mTOR kinase domain with docked XL765. *A*. The mTOR kinase domain and XL765 are depicted in surface and red sticks representations. The binding site is blown-up and the interacting residues are labeled and presented in surface colored differently. *B-C*: Comparative binding analysis of XL765 (A) and the native ligand PI-103 (B). The hydrogen bonds are presented as green-dashed lines labeled with bond lengths and the residues participating in non-bonding interactions are presented as red arcs. The common interacting residues for both the ligands are encircled.

Molecular docking study of XL765 with mTOR showed that the drug in the catalytic site was found rapped by 11 residues including Ile-2163, Leu-2185, Trp-2239, Val-2240, Asp-2244, Thr-2245, Ala-2248, Arg-2251, Asp-2252, Met-2345, and Ile-2356 (Fig 3, Table 3). The residue Asp-2251 was engaged in hydrogen bonding using N-amino atom of guanidium group to one of the N-atom of quinoxaline moiety of XL765 and all the interacting residues exerted 37 non-bonding interactions making the drug-protein complex stabilized (Fig 3B, Table 2). The strength of XL765 binding was evident from high absolute values of the dock score, binding energy, and dissociation constant (Table 2). The residue Trp-2239 was pinpointed as the key interacting residue as it observed maximum ΔASA (56.39 Å^2^) and was also participating in maximum number of 10 non-bonding interactions. Further, the Trp-2239 was also seeming to be involved in aromatic interaction with the terminal benzene ring of bulkier group attached to quinoxaline moiety of XL765.

**Table 3:** The mTOR residues interacting with XL765 are listed with the number of non-bonding interactions and ΔASA. The residue participating in hydrogen bond formation is indicated as ‘H-bond’ in parentheses with the residue name.

Interacting residues	Non-bonding interactions	ΔASA (Å^2^)
Ile-2163	2	27.24
Leu-2185	1	25.32
Trp-2239	10	56.39
Val-2240	1	8.9
Asp-2244	3	28.52
Thr-2245	1	27.82
Ala-2248	1	16.09
Arg-2251 (H-bond)	6	55.76
Asp-2252	3	24.43
Met-2345	6	30.62
Ile-2356	3	31.64

On comparing the docked XL765 with the native ligand PI-103, five residues Ile-2163, Leu-2185, Trp-2239, Val-2240, and Ile-2356 were identified as common interacting residues for both the ligands (Fig 3B and 3C). Of these common interacting residues, the residue Trp-2239 was marked as the key residue in XL765 binding. Another common interacting residue, Val-2240 formed a hydrogen bond with the native ligand, however in case of XL765, it was involved in non-bonding interaction only. These findings showed the relevance of the common interacting residues in binding and thus, XL765 engaged the important interacting residues in binding like that of the native ligand and inhibited mTOR in the similar way [44–45].

### Combinatorial library generation for XL765 scaffold

**Fig 4:**
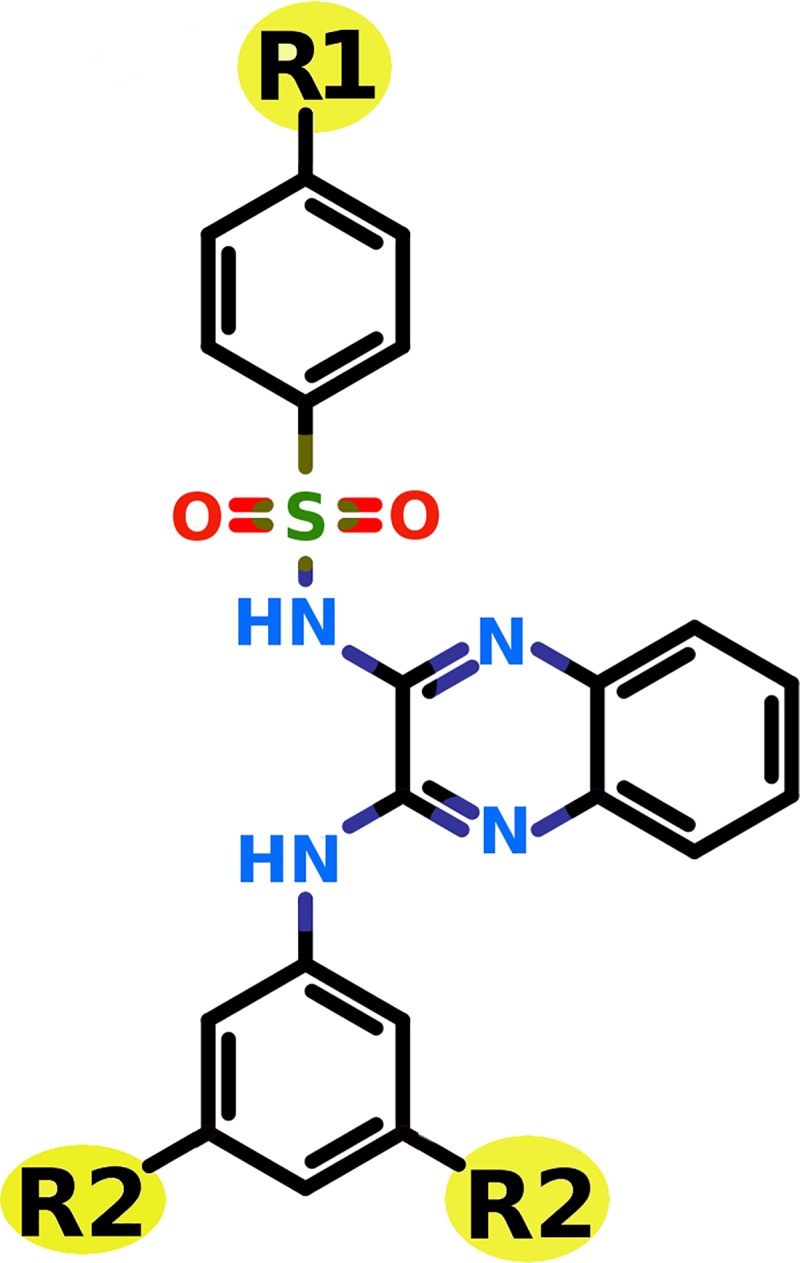
Two dimensional structure of the scaffold from XL765 considered for combinatorial library generation. The R_1_ and R_2_ are the substiuent groups used to modify the scaffold to generate combinatorial library.

**Fig 5:**
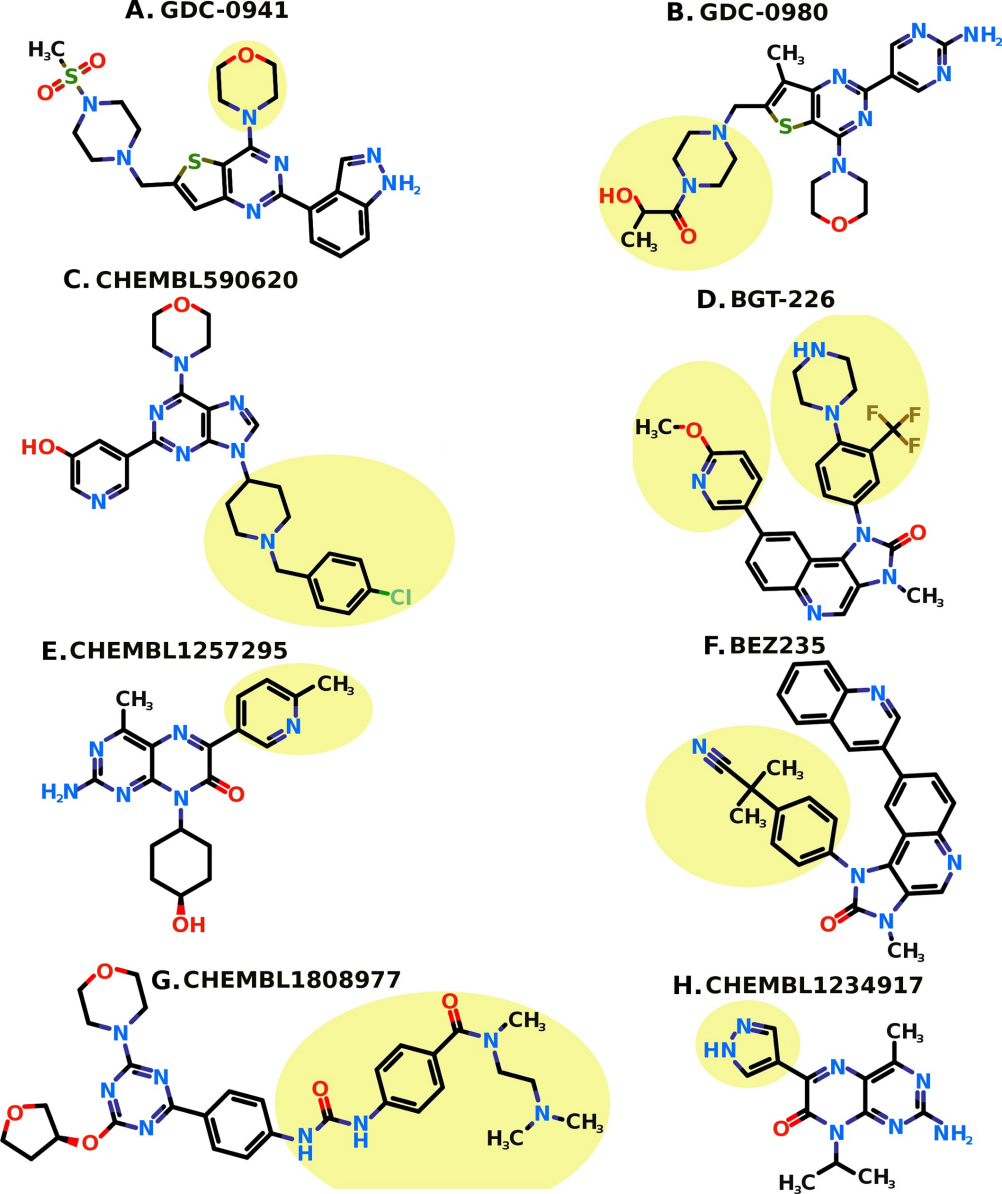
The known PI3K/mTOR inhibitors with the highlighted portions in light yellow. The nine highlighted portions are selected as R1 substituent groups from the known eight inhibitors.

Keeping the scaffold intact from XL765 and replacement of R_1_ and R_2_ with substituents (Fig 4) generated a library of compounds. The nine substituents were considered for R_1_ replacement from the previous known PI3K/mTOR inhibitors (Fig 5) [65]. Whereas 5 small substituents including ‘-O-CH_3_’, ‘-OH’, ‘-NH_2_’, ‘-F’, and ‘-Br’ were used for R_2_ replacement. These R_1_ and R_2_ replacements generate a library of total 45 compounds used in this study for virtual screening against PI3K*γ* and mTOR. The compounds generated were named as sequential numbers from 1 to 45 in the order of systematic substitution of R_1_ and R_2_ groups (S1 Table).

### Virtual screening of generated combinatorial library

The combinatorial library of all the 45 compounds used for virtual screening and their dock scores for PI3K*γ* and mTOR is provided as S1 Table. The six compounds were found common among top 20 scoring compounds of PI3K*γ* and mTOR (Fig 1B–1G, Table 2). In general, these six compounds have better dock score, binding energy, and dissociation constant than XL765. These compounds were proposed as potential dual PI3K/mTOR inhibitors and further checked for drug-likeness and pharmacokinetic properties prediction and enrichment evaluation analysis for docking accuracy.

### Drug-likeness and pharmacokinetic properties

The values for all 5 conditions (rule of 5) for the proposed inhibitors were better than that of XL765 (Table 4). XL765 was having undesired molecular weight (> 500), while all the proposed inhibitors have desired molecular weight less than 500 dalton except for two compounds 38 & 9. The lipophilicity (LogP) value of XL765 has undesired value (>5), while all the proposed inhibitors have desired LogP values within the range of five.

**Table 4:** Drug-likeness (Lipinski rule of five) for XL765 and the six proposed dual inhibitors 28, 18, 38, 9, 10, and 19.

Lipinski rule of five								
Property	Desired value	XL765	28	18	38	9	10	19
Mol Wt.	<500	599.67	497.53	473.49	690.42	501.55	493.55	491.58
H-Bond Donors	<5	3	2	4	3	2	4	4
H-Bond Acceptors	<10	9	7	8	8	8	9	9
Rotatable Bonds	<10	10	6	6	7	8	6	6
Lipophilicity (LogP)	<5	5.76	4.29	3.53	4.73	4.14	3.42	3.18

While comparing pharmacokinetic properties (absorption, distribution, metabolism, excretion, and toxicity), the proposed inhibitors passed most of the tests and were comparable to XL765 (Table 5). Therefore, this study proposed the six proposed inhibitors as safe drug candidates for treatment in humans.

**Table 5:** Pharmacokinetic properties (ADMET) prediction for XL765 and the six proposed dual inhibitors 28, 18, 38, 9, 10, and 19.

Pharmacokinetic properties (ADMET)										
Property	Model Name	Desired value	Unit	XL765	28	18	38	9	10	19
ABSORPTION	Water solubility		log mol/L	-3.49	-4.40	-3.31	-4.07	-4.41	-3.32	-3.31
	Caco2 permeability	>0.90	log Papp in 10–6 cm/s	0.37	0.47	0.42	0.22	0.16	0.39	0.45
	Intestinal absorption (human)	>>30	% Absorbed	100	100	90.49	80.08	96.11	89.09	82.79
	Skin Permeability	>-2.5	log Kp	-2.73	-2.73	-2.73	-2.73	-2.73	-2.73	-2.73
	P-glycoprotein substrate	No	Yes/No	No	Yes	Yes	Yes	Yes	Yes	Yes
	P-glycoprotein I inhibitor		Yes/No	Yes	Yes	Yes	Yes	Yes	Yes	Yes
	P-glycoprotein II inhibitor		Yes/No	Yes	Yes	Yes	Yes	Yes	Yes	Yes
DISTRIBUTION	VDss (human)	0.71<VDss<2.81	log L/kg	-1.56	-0.82	-0.90	-0.61	-0.79	-0.65	-0.61
	Fraction unbound (human)		Fu	0.14	0	0	0	0	0	0
	BBB permeability	<0.3	log BB	-1.62	-0.42	-1.45	-1.39	-0.44	-1.51	-1.32
	CNS permeability	>-2	log PS	-3.42	-3.32	-3.47	-3.38	-3.32	-3.62	-2.83
METABOLISM	CYP2D6 substrate	No	Yes/No	No	No	No	No	No	No	No
	CYP3A4 substrate	No	Yes/No	Yes	Yes	Yes	Yes	Yes	Yes	Yes
	CYP1A2 inhibitor		Yes/No	No	No	No	No	No	No	No
	CYP2C19 inhibitor		Yes/No	No	Yes	No	Yes	Yes	No	No
	CYP2C9 inhibitor		Yes/No	Yes	Yes	Yes	Yes	Yes	Yes	Yes
	CYP2D6 inhibitor		Yes/No	No	No	No	No	No	No	No
	CYP3A4 inhibitor		Yes/No	Yes	Yes	Yes	Yes	Yes	Yes	Yes
EXCRETION	Total Clearance		log ml/min/kg	0.03	-0.18	0.18	-0.13	0.36	0.07	-0.12
	Renal OCT2 substrate	No	Yes/No	No	No	No	No	No	No	No
TOXICITY	AMES toxicity	No	Yes/No	No	No	No	No	No	No	No
	Max. tolerated dose (human)	<0.477	log mg/kg/day	0.34	0.22	0.52	0.33	0.18	0.56	0.56
	hERG I inhibitor	No	Yes/No	No	No	No	No	No	No	No
	hERG II inhibitor	No	Yes/No	Yes	Yes	Yes	Yes	Yes	Yes	Yes
	Oral Rat Acute Toxicity (LD50)		mol/kg	3.16	2.68	2.73	2.80	2.66	2.71	2.53
	Oral Rat Chronic Toxicity (LOAEL)		log mg/kg_bw/day	2.14	1.65	2.75	1.88	1.68	2.72	2.58
	Hepatotoxicity	No	Yes/No	Yes	Yes	Yes	Yes	Yes	Yes	Yes
	Skin Sensitization	No	Yes/No	No	No	No	No	No	No	No
	*T*. *pyriformis* toxicity	<-0.5	log ug/L	0.28	0.28	0.28	0.28	0.28	0.28	0.28
	Minnow toxicity	>-0.3	log mM	0.52	0.91	0.83	-0.10	0.09	1.56	0.99

ADMET, absorption, distribution, metabolism, excretion, and toxicity; BBB, blood‐brain barrier; CNS, central nervous system; CYP, cytochrome P; hERG, human ether-a-go-go-related gene; LD50, lethal dose 50%; LOAEL, lowest observed adverse effect level; OCT2, organic cation transporter 2; VDss, steady-state volume of distribution.

### Enrichment evaluation analysis

**Fig 6:**
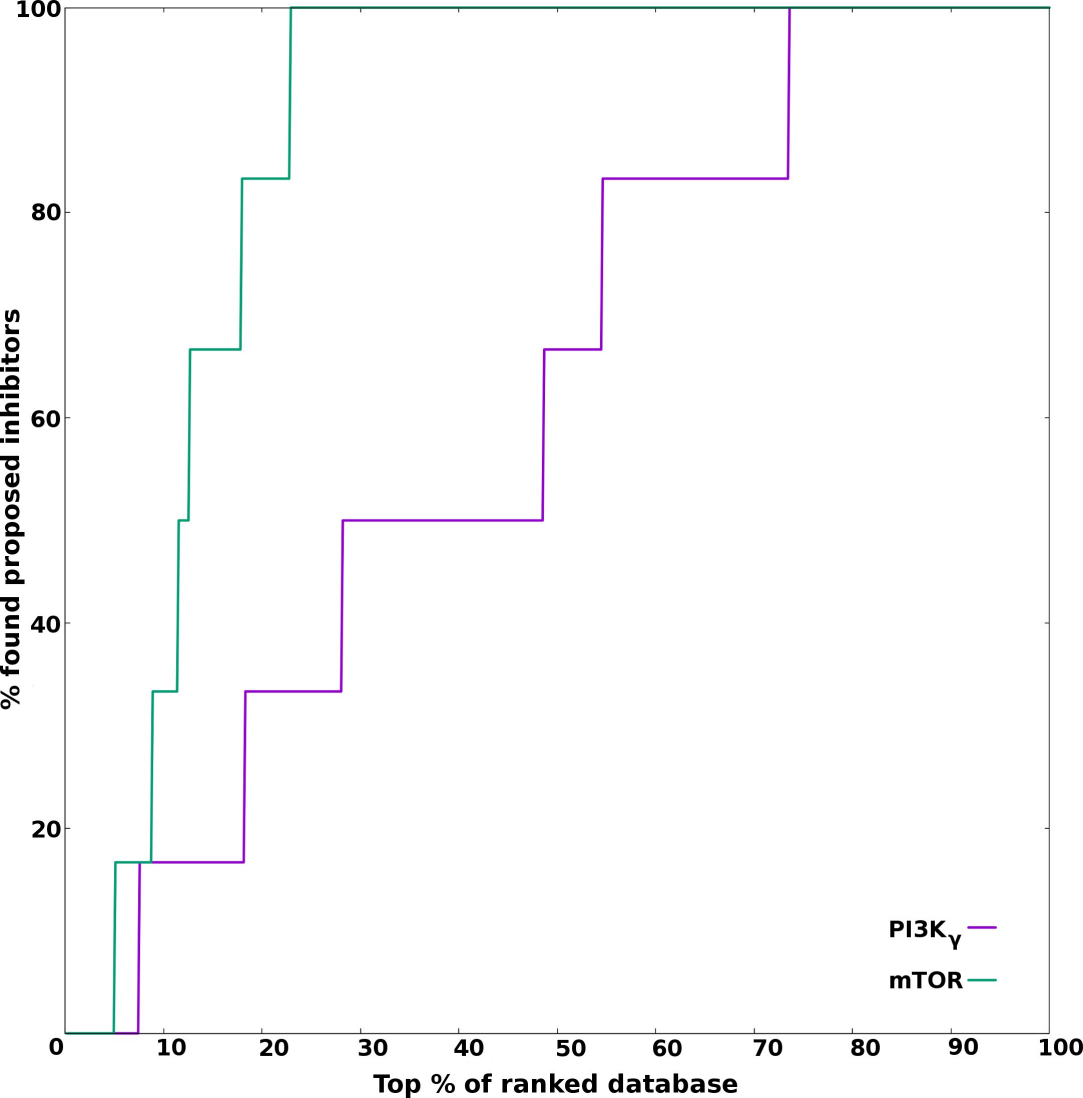
Enrichment curve for the proposed inhibitors. The curves for the two targets PI3Kγ and mTOR are shown by purple and green colors respectively.

For enrichment analysis, 100 decoys were generated for each compound and thus, making the count to 600 for the six proposed inhibitors. These 600 decoys along with six proposed inhibitors were screened using molecular docking against molecular targets PI3Kγ and mTOR. The enrichment analysis of the screening is shown in Fig 6. The complete random selection of proposed inhibitors will yield EF = 1. An EF = 5 means proposed inhibitors were observed five times more in the top 20% of the ranked database than observed in random 20% sampling of the database. When the sample subsetting level is set at 20%, the theoretical maximum that the enrichment factor achieve is 20. In the current study, the EF values at 20% subsetting level were 6.67 and 16.67 for PI3Kγ and mTOR respectively. The enrichment analysis for the target PI3Kγ revealed that the five of the six proposed inhibitors were picked in the top 55% of the screened database of decoys and proposed inhibitors. While for the target mTOR, all the six proposed inhibitors were picked in the top 23% of the screened database. Thus, the enrichment analysis showed good enrichment for mTOR, while modest enrichment for PI3K. However, the current work explored dual PI3K/mTOR inhibitors and thus sought common compounds in top 20 scoring for both the targets, and thus may compensate for modest docking accuracy of PI3K. Overall, the enrichment analysis suggest that the six proposed inhibitors were selectively picked over the decoys dataset by virtual screening procedure for both the targets.

### Comparative binding analyses of six proposed dual inhibitors for PI3Kγ

**Fig 7:**
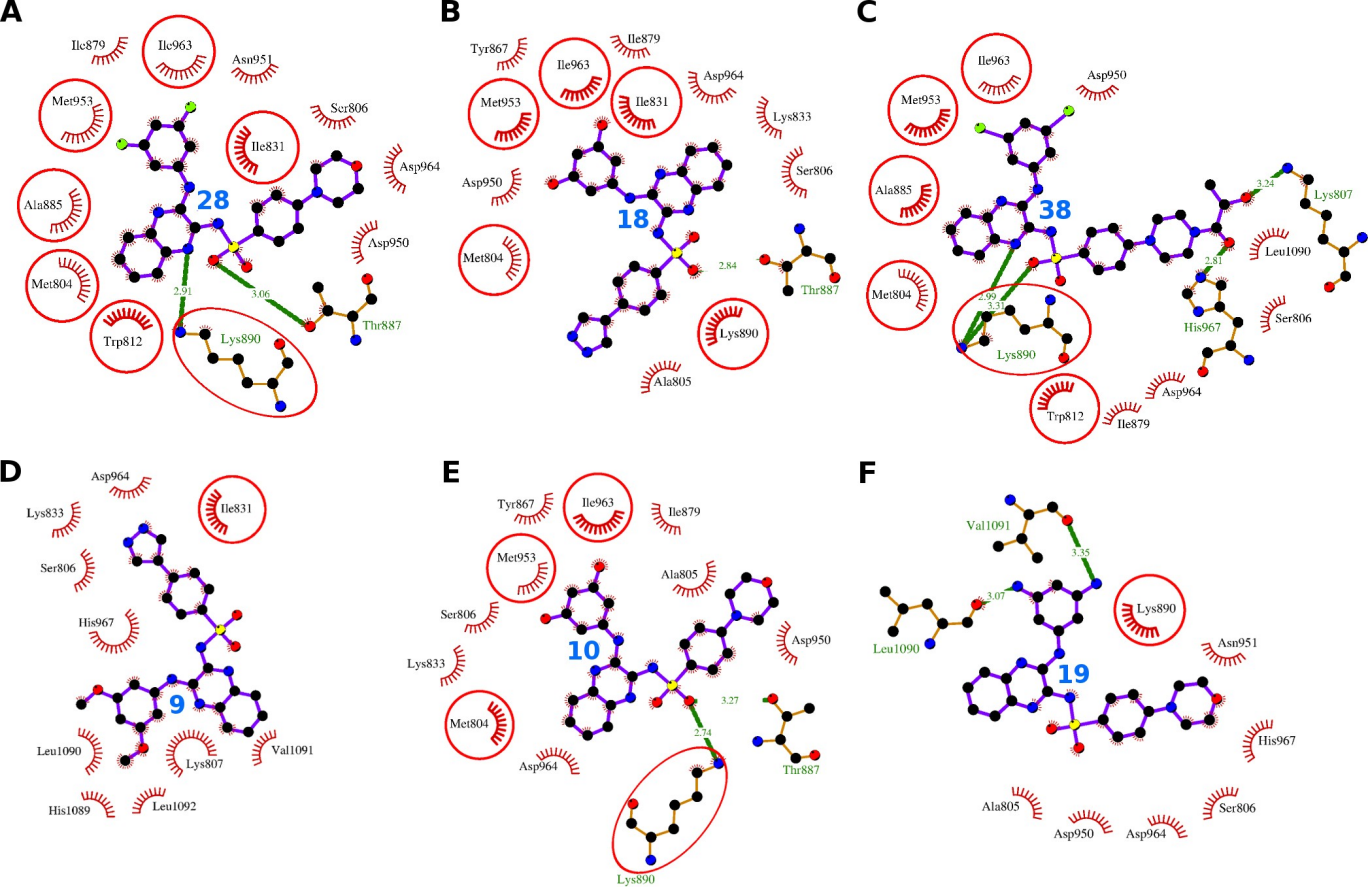
Comparative PI3Kγ binding analysis of the compounds 28, 18, 38, 9, 10, and 19. The hydrogen bonds are presented as green-dashed lines labeled with bond lengths and the residues participating in non-bonding interactions are presented as red arcs. The interacting residues common with the interacting residues of XL765 are encircled.

The compound 28 was identified as the compound with highest dock score (-48.59) in PI3K*γ* docking (Table 2). It bound to the catalytic site and interact with 13 residues Met-804, Ser-806, Trp-812, Ile-831, Ile-879, Ala-885, Thr-887, Lys-890, Asp-950, Asn-951, Met-953, Ile-963, and Asp-964 (Fig 7, Table 6). These 13 residues formed 37 non-bonding interactions and the two residues Thr-887 and Lys-890 formed 2 hydrogen bonding interactions (S2 Table). The absolute values of dock score (-48.59), binding energy (-9.14), and pK_d_ (6.70) were greater than that of XL765 (Table 2).

**Table 6:** Interacting residues of PI3Kγ for the compounds XL765, 28, 18, 38, 9, 10, 19, and native ligand. Each column has interacting residues of PI3Kγ for a particular compound whose name is mentioned in bold at the top in the first row. The interacting residues common with those of XL765 are shown in bold and italics.

XL765	28	18	38	9	10	19	Native
Lys-802	-	-	-	-	-	-	-
***Met-804***	***Met-804***	***Met-804***	***Met-804***	***-***	***Met-804***	***-***	***Met-804***
-	-	Ala-805	-	-	Ala-805	Ala-805	-
-	Ser-806	Ser-806	Ser-806	Ser-806	Ser-806	Ser-806	-
-	-	-	Lys-807	Lys-807	-	-	-
***Trp-812***	***Trp-812***	***-***	***Trp-812***	***-***	***-***	***-***	***Trp-812***
***Ile-831***	***Ile-831***	***Ile-831***	***-***	***Ile-831***	***-***	***-***	***Ile-831***
-	-	Lys-833	-	Lys-833	Lys-833	-	Lys-833
-	-	Tyr-867	-	-	Tyr-867	-	Tyr-867
-	Ile-879	Ile-879	Ile-879	-	Ile-879	-	Ile-879
-	-	-	-	-	-	-	Glu-880
-	-	-	-	-	-	-	Ile-881
***Val-882***	***-***	***-***	***-***	***-***	***-***	***-***	***Val-882***
***Ala-885***	***Ala-885***	***-***	***Ala-885***	***-***	***-***	***-***	***-***
Thr-886	-	-	-	-	-	-	-
-	Thr-887	Thr-887	-	-	Thr-887	-	Thr-887
***Lys-890***	***Lys-890***	***Lys-890***	***Lys-890***	***-***	***Lys-890***	***Lys-890***	***-***
-	Asp-950	Asp-950	Asp-950	-	Asp-950	Asp-950	-
-	Asn-951	-	-	-	-	Asn-951	-
***Met-953***	***Met-953***	***Met-953***	***Met-953***	***-***	***Met-953***	***-***	***Met-953***
***Ile-963***	***Ile-963***	***Ile-963***	***Ile-963***	***-***	***Ile-963***	***-***	***Ile-963***
-	Asp-964	Asp-964	Asp-964	Asp-964	Asp-964	Asp-964	Asp-964
-	-	-	His-967	His-967	-	His-967	-
-	-	-	-	His-1089	-	-	-
-	-	-	Leu-1090	Leu-1090	-	Leu-1090	-
-	-	-	-	Val-1091	-	-	-
-	-	-	-	Leu-1092	-	-	-

The compound 18 interacted with 13 residues in the catalytic site including Met-804, Ala-805, Ser-806, Ile-831, Lys-833, Tyr-867, Ile-879, Thr-887, Lys-890, Asp-950, Met-953, Ile-963, and Asp-964 (Fig 7, Table 6). These 13 residues exerted 45 non-bonding interactions and one hydrogen bond through the residue Thr-887 (S3 Table). The high absolute values of dock score (-45.12), binding energy (-8.49), and dissociation constant (6.23) further provide weight to the tight binding to the protein (Table 2). The Asp-964 turned out to be the most important interacting residue as showed maximum ΔASA (45.35 Å^2^) and involved in maximum of 16 non-bonding interactions.

The compound 38 bound to the catalytic site using 13 interacting residues Met-804, Ser-806, Lys-807, Trp-812, Ile-879, Ala-885, Lys-890, Asp-950, Met-953, Ile-963, Asp-964, His-967, and Leu-1090 (Fig 7, Table 6). These 13 residues formed 37 non-bonding interactions and four hydrogen bond. The residues Lys-807, Lys-890, and His-967 formed one, two, and one hydrogen bonds respectively (S4 Table). The absolute value of dock score (-42.95) was greater than that of XL765, whereas the binding energy (-9.98) and dissociation constants (7.32) were comparable (Table 2).

The compound 9 bound to the catalytic site through 10 interacting residues Ser-806, Lys-807, Ile-831, Lys-833, Asp-964, His-967, His-1089, Leu-1090, Val-1091, and Leu-1092 (Fig 7, Table 6). These 10 residues formed 38 non-bonding interactions stabilized the protein-ligand complex (S5 Table). The high absolute values of dock score (-39.45), binding energy (-7.82), and dissociation constant (5.73) indicated towards quality docking (Table 2). The Lys-807 was proposed to be most important interacting residue as it observed maximum ΔASA (76.04 Å^**2**^) and participated in maximum of 12 non-bonding interactions.

The compound 10 bound to the catalytic site interacting with 11 residues including Thr-887, Met-804, Ala-805, Ser-806, Lys-833, Tyr-867, Ile-879, Lys-890, Asp-950, Met-953, Ile-963, and Asp-964 (Fig 7, Table 6). These 11 residues form 52 non-bonding interactions and two hydrogen bonds (S6 Table). The high absolute values of dock score (-34.44), binding energy (-8.49), and dissociation constant (6.23) indicated towards quality docking (Table 2). The Lys-890 and Asp-964 were turned out to be most important interacting residues as these were involved in maximum of 11 non-bonding interactions each and observed two maxima of ΔASA, 47.69 Å^**2**^ and 47.03 Å^**2**^ respectively.

The compound 19 interacting with eight residues (Val-1091, Ala-805, Ser-806, Lys-890, Asp-950, Asn-951, Asp-964, His-967, and Leu-1090) in the catalytic site forming 24 non-bonding interactions and two hydrogen bonds (Fig 7, Table 6, S7 Table). The high absolute values of dock score (-38.32), binding energy (-7.45), and dissociation constant (5.46) indicated towards quality docking (Table 2).

Summing up, all the six compounds showed similar binding pattern to that of XL765 and the binding scores were also comparable. The two residues Asp-964 and Ser-806 were commonly found as interacting residues for all the six compounds and one residue Asp-964 was consistently appearing as interacting residue in all the six compounds and the native ligand.

## Comparative binding analyses of six proposed dual inhibitors for mTOR

**Fig 8:**
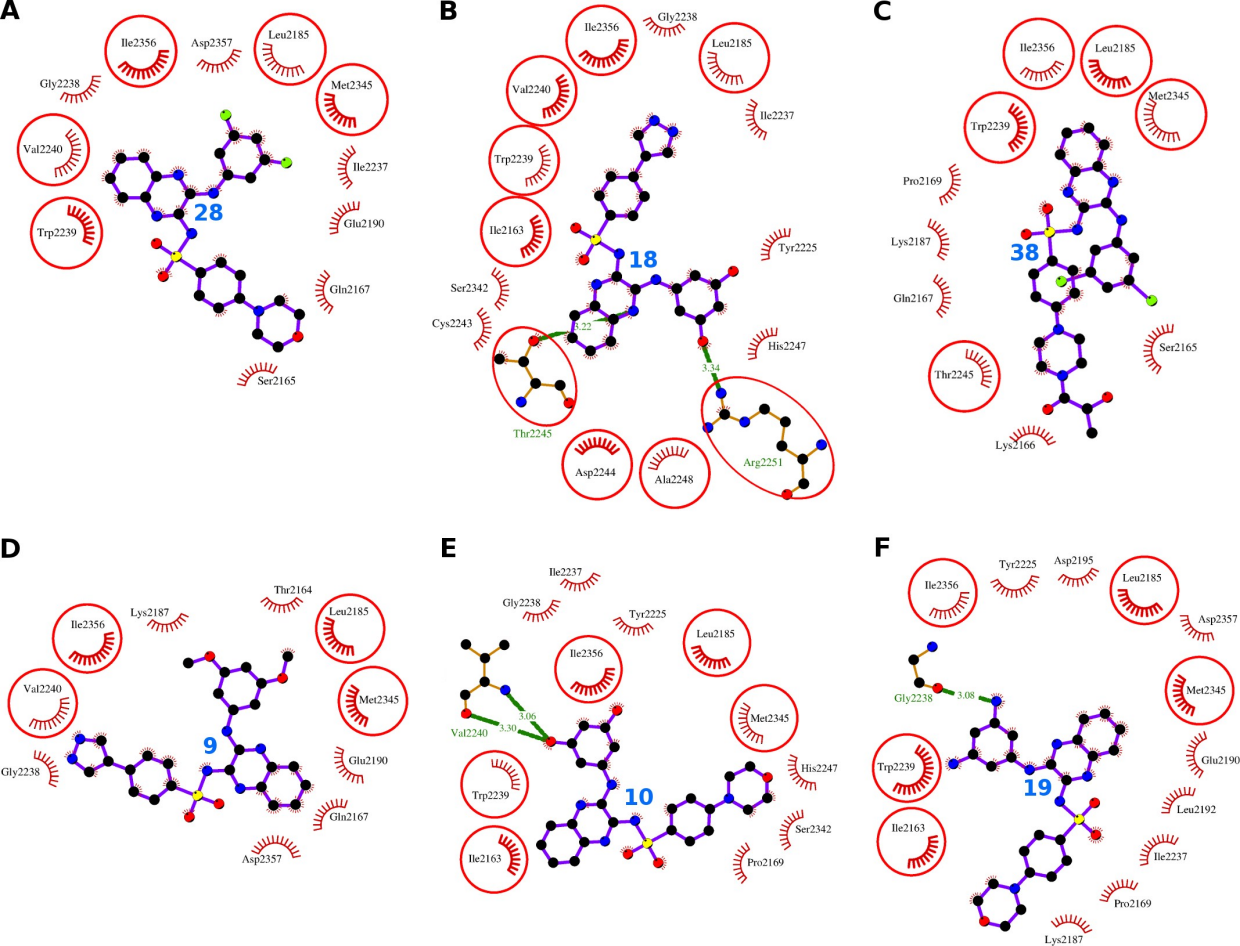
Comparative mTOR binding analysis of the compounds 28, 18, 38, 9, 10, and 19. The hydrogen bonds are presented as green-dashed lines labeled with bond lengths and the residues participating in non-bonding interactions are presented as red arcs. The interacting residues which are common with the interacting residues of XL765 are encircled.

The compound 28 binds to the catalytic site of mTOR lined by 11 interacting residues Ser-2165, Gln-2167, Leu-2185, Glu-2190, Ile-2237, Gly-2238, Trp-2239, Val-2240, Met-2345, Ile-2356, and Asp-2357 (Fig 8, Table 7). These 11 interacting residues formed 31 non-bonding interactions, and thus stabilized the complex of the ligand and the protein (Table 2). Further, the values of dock score (-48.32), binding energy (-9.16), and dissociation constant (6.71) provide weight to the good quality binding (Table 2). The residues Ile-2356 and Asp-2357 were pinpointed as most importanct interacting residues as these showed two maximum ΔASA 44.74 Å^2^ and 46.01 Å^2^, and number of non-bonding interactions 7 and 8 respectively (S8 Table).

**Table 7:** Interacting residues of mTOR for the compounds XL765, 28, 18, 38, 9, 10, 19, and native ligand. Each column has interacting residues of mTOR for a particular compound whose name is mentioned in bold at the top in the first row. The interacting residues common with those of XL765 are shown in bold and italics.

XL765	28	18	38	9	10	19	Native
***Ile-2163***	***-***	***Ile-2163***	***-***	***-***	***Ile-2163***	***Ile-2163***	***Ile-2163***
-	-	-	-	Thr-2164	-	-	-
-	Ser-2165	-	Ser-2165	-	-	-	-
-	-	-	Lys-2166	-	-	-	-
-	Gln-2167	-	Gln-2167	Gln-2167	-	-	-
-	-	-	Pro-2169	-	Pro-2169	Pro-2169	-
***Leu-2185***	***Leu-2185***	***Leu-2185***	***Leu-2185***	***Leu-2185***	***Leu-2185***	***Leu-2185***	***Leu-2185***
-	-	-	Lys-2187	Lys-2187	-	Lys-2187	-
-	Glu-2190	-	-	Glu-2190	-	Glu-2190	Glu-2190
-	-	-	-	-	-	Leu-2192	-
-	-	-	-	-	-	Asp-2195	Asp-2195
-	-	Tyr-2225	-	-	Tyr-2225	Tyr-2225	Tyr-2225
-	Ile-2237	Ile-2237	-	-	Ile-2237	Ile-2237	Ile-2237
-	Gly-2238	Gly-2238	-	Gly-2238	Gly-2238	-	Gly-2238
***Trp-2239***	***Trp-2239***	***Trp-2239***	***Trp-2239***	***-***	***Trp-2239***	***Trp-2239***	***Trp-2239***
***Val-2240***	***Val-2240***	***Val-2240***	***-***	***Val-2240***	***-***	***-***	***Val-2240***
-	-	Cys-2243	-	-	-	-	-
***Asp-2244***	***-***	***Asp-2244***	***-***	***-***	***-***	***-***	***-***
***Thr-2245***	***-***	***Thr-2245***	***Thr-2245***	***-***	***-***	***-***	***-***
-	-	His-2247	-	-	His-2247	-	-
***Ala-2248***	***-***	***Ala-2248***	***-***	***-***	***-***	***-***	***-***
***Arg-2251***	***-***	***Arg-2251***	***-***	***-***	***-***	***-***	***-***
Asp-2252	-	-	-	-	-	-	-
-	-	Ser-2342	-	-	Ser-2342	-	-
***Met-2345***	***Met-2345***	***-***	***Met-2345***	***Met-2345***	***Met-2345***	***Met-2345***	***-***
***Ile-2356***	***Ile-2356***	***Ile-2356***	***Ile-2356***	***Ile-2356***	***Ile-2356***	***Ile-2356***	***Ile-2356***
-	Asp-2357	-	-	Asp-2357	-	Asp-2357	Asp-2357

The compound 18 found to bind in catalytic site interacting with 15 residues Ile-2163, Leu-2185, Tyr-2225, Ile-2237, Gly-2238, Trp-2239, Val-2240, Cys-2243, Asp-2244, Thr-2245, His-2247, Ala-2248, Arg-2251, Ser-2342, and Ile-2356 (Fig 8, Table 7). These 15 residues formed 39 non-bonding interactions and 2 hydrogen bonds (Table 2). The two hydrogen bonds were formed by the residues Thr-2245 and Arg-2251 respectively (S9 Table). The binding scores including dock score (-46.96), binding energy (-8.80), and dissociation constant (6.45) were comparable to other similar compounds (Table 2). While Ile-2356 was pinpointed as the most important interacting residue as it observed maximum ΔASA (44.7 Å^2^) and was involved in 5 non-bonding interactions.

The compound 38 bound to the catalytic site and interacts with 10 residues including Ser-2165, Lys-2166, Gln-2167, Pro-2169, Leu-2185, Lys-2187, Trp-2239, Thr-2245, Met-2345, and Ile-2356 (Fig 8, Table 7). These 10 residues form 31 non-bonding interactions and stabilize the complex. The values of the dock score (-44.91), the binding energy (-8.82), and the dissociation constant (6.47) were also comparable to that of other similar compounds and XL765 (Table 2). The residue Trp-2239 was pinpointed as the most important residue as it observed maximum ΔASA (44.09 Å^2^) and was participating in maximum number of seven non-bonding interactions (S10 Table).

The compound 9 sits in the catalytic site and interact with 10 residues Thr-2164, Gln-2167, Leu-2185, Lys-2187, Glu-2190, Gly-2238, Val-2240, Met-2345, Ile-2356, and Asp-2357 (Fig 8, Table 7). These 10 interacting residues exerted 30 non-bonding interactions and stabilized the protein-ligand complex (Table 2). The values of the dock score (-46.26), binding energy (-8.23), and dissociation constant (6.03) were also comparable to that of similar compounds (Table 2). The residue Ile-2356 was proposed as most important interacting residue as it observed maximum ΔASA (44.42 Å^2^) and participated in maximum number of five non-bonding interactions (S11 Table).

The compound 10 bound to the catalytic site and interacted with 11 residues Val-2240, Ile-2163, Pro-2169, Leu-2185, Tyr-2225, Ile-2237, Gly-2238, Trp-2239, His-2247, Ser-2342, Met-2345, and Ile-2356 (Fig 8, Table 7). These 11 residues exerted 26 non-bonding interactions and the two hydrogen bonds through single residue Val-2240 (S12 Table). The values of the dock score (-45.91), binding energy (-8.71), and dissociation constant (6.39) were also comparable to that of similar compounds (Table 2). The residue Trp-2239 was proposed as most important interacting residue as it observed maximum ΔASA (46.27 Å^2^) and was engaged in maximum of six non-bonding interactions (S12 Table).

The compound 19 interacted with 13 residues including Gly-2238, Ile-2163, Pro-2169, Leu-2185, Lys-2187, Glu-2190, Leu-2192, Asp-2195, Tyr-2225, Ile-2237, Trp-2239, Met-2345, Ile-2356, and Asp-2357 (Fig 8, Table 7). These 13 residues formed 42 non-bonding interactions and one hydrogen bond through Gly-2238 (S13 Table). The values of the dock score (-43.70), binding energy (-9.46), and dissociation constant (6.94) were also comparable to that of similar compounds (Table 2). The residue Trp-2239 was proposed as most important interacting residue as it observed maximum ΔASA (49.03 Å^2^) and was engaged in maximum number of six non-bonding interactions (S13 Table).

To sum up, all the four compounds were showing similar binding patterns with comparable binding scores. The two residues Ile-2356 and Leu-2185 were consistently appearing as interacting residues for all the six compounds and the native ligand.

## Conclusions

The current study explored the binding pose and the molecular interactions of dual inhibitor XL765 with PI3Kγ and mTOR using molecular docking analyses. The binding pose of XL765 with various interacting residues were determined and characterized. Among XL765 interacting residues of PI3Kγ, Lys-890 and Met-953 were pinpointed as the key residues required for binding. Whereas in case of mTOR, the Trp-2239 was pinpointed as the key interacting residue and another residue Asp-2251 contributed a hydrogen-bonding interaction using N-amino atom of guanidium group to one of the N-atom of quinoxaline moiety of XL765. The virtual screening of combinatorial library generated by modification of scaffold considered from XL765 led to identification of six novel compounds. The compounds passes most of the tests in drug-likeness and pharmacokinetic properties evaluation, which suggest that the six novel compounds can be used as safe drug candidates for treatment in humans. In addition to the better binding scores, the enrichment analyses also prove the selective and quality binding to the targets PI3Kγ and mTOR. The detailed and comparative analyses with XL765 indicated these six novel compounds as better dual PI3K/mTOR inhibitors than the starting compound XL765. Thus, the present docking analyses of dual inhibitor XL765 with PI3Kγ and mTOR will provide an excellent model for studying molecular interactions of drug-protein complex where the drug is targeting multiple proteins and will also help in designing the novel and efficacious drugs.

## Supporting information

S1 Table The compounds generated are named as sequential numbers from 1 to 45 in the order of systematic substitution of R1 and R2 groups. The compounds are provided with structure of varying R_1_ and R_2_ groups, and dock scores for PI3Kγ and mTOR docking. The selected six compounds are shown in bold.
(DOC)

S2 Table The human PI3Kγ residues interacting with compound 28 are listed with the number of hydrogen bonds, number of non-bonding interactions, and ΔASA. (DOC)

S3 Table The human PI3Kγ residues interacting with compound 18 are listed with the number of hydrogen bonds, number of non-bonding interactions, and ΔASA. (DOC)

S4 Table The human PI3Kγ residues interacting with compound 38 are listed with the number of hydrogen bonds, number of non-bonding interactions, and ΔASA. (DOC)

S5 Table The human PI3Kγ residues interacting with compound 9 are listed with the number of hydrogen bonds, number of non-bonding interactions, and ΔASA. (DOC)

S6 Table The human PI3Kγ residues interacting with compound 10 are listed with the number of hydrogen bonds, number of non-bonding interactions, and ΔASA. (DOC)

S7 Table The human PI3Kγ residues interacting with compound 19 are listed with the number of hydrogen bonds, number of non-bonding interactions, and ΔASA. (DOC)

S8 Table The human mTOR residues interacting with compound 28 are listed with the number of hydrogen bonds, number of non-bonding interactions, and ΔASA. (DOC)

S9 Table The human mTOR residues interacting with compound 18 are listed with the number of hydrogen bonds, number of non-bonding interactions, and ΔASA. (DOC)

S10 Table The human mTOR residues interacting with compound 38 are listed with the number of hydrogen bonds, number of non-bonding interactions, and ΔASA. (DOC)

S11 Table The human mTOR residues interacting with compound 9 are listed with the number of hydrogen bonds, number of non-bonding interactions, and ΔASA. (DOC)

S12 Table The human mTOR residues interacting with compound 10 are listed with the number of hydrogen bonds, number of non-bonding interactions, and ΔASA. (DOC)

S13 Table The human mTOR residues interacting with compound 19 are listed with the number of hydrogen bonds, number of non-bonding interactions, and ΔASA. (DOC)

## Abbreviations

PI3K: phosphatidylinositide 3-kinase
PIP2: phosphatidylinositol-4,5-bisphosphate
PIP3: phosphatidylinositol-3,4,5-trisphosphate
mTOR: mammalian target of rapamycin
mTORC1: mTOR complex 1
mTORC2: mTOR complex 2
ASA: accessible surface area
ΔASA: loss in accessible surface area
PI3Kγ: class-IA PI3K p110γ
