# Supplementary material for: Anticancer compound XL765 as PI3K/mTOR dual inhibitor: A structural insight into the inhibitory mechanism using computational approaches
Source: PLoS One. 2019 Jun 27;14(6):e0219180. doi: 10.1371/journal.pone.0219180 (PMC6597235; doi:10.1371/journal.pone.0219180)
Supplement: S1 Table — The compounds are provided with structure of varying R1 and R2 groups, and dock scores for PI3Kγ and mTOR docking. The selected six compounds are shown in bold. (DOC) [file pone.0219180.s001.doc]

S1 Table. The compounds generated are named as sequential numbers from 1 to 45 in the order of systematic substitution of R_1_ and R_2_ groups. The compounds are provided with structure of varying R_1_ and R_2_ groups, and Dock Scores for PI3K and mTOR docking. The selected six compounds are shown in bold.

| **Compd** | **R_1_** | **R_2_** | **PI3K Dock Score** | **mTOR Dock Score** |
| --- | --- | --- | --- | --- |
| 1 | 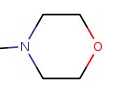 | 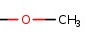 | -27.38 | -48.55 |
| 2 | 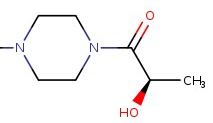 | 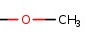 | -40.62 | -26.76 |
| 3 | 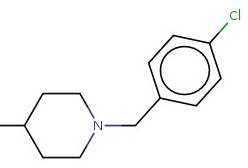 | 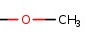 | -19.19 | -53.33 |
| 4 | 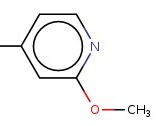 | 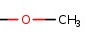 | -38.72 | -38.82 |
| 5 | 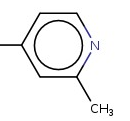 | 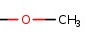 | -41.59 | -42.17 |
| 6 | 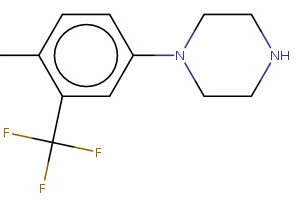 | 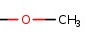 | -33.13 | -36.58 |
| 7 | 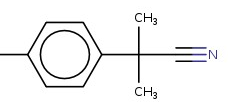 | 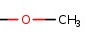 | -32.91 | -46.95 |
| 8 | 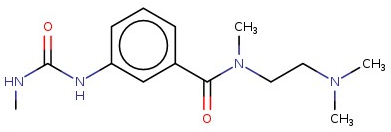 | 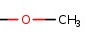 | -31.70 | -38.01 |
| **9** | **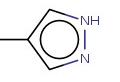** | **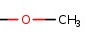** | **-39.45** | **-46.27** |
| **10** | **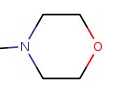** | **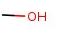** | **-34.44** | **-45.92** |
| 11 | 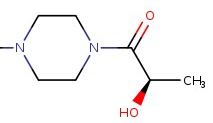 | 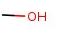 | -45.71 | -40.81 |
| 12 | 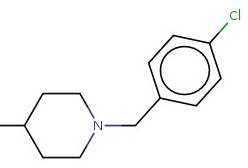 | 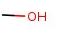 | -24.50 | -36.45 |
| 13 | 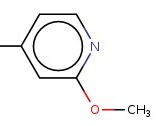 | 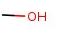 | -41.27 | -36.45 |
| 14 | 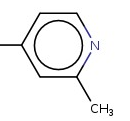 | 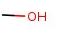 | -39.58 | -27.60 |
| 15 | 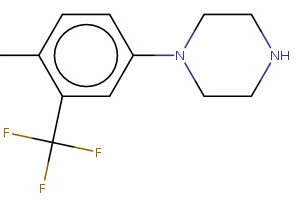 | 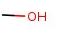 | -32.20 | -50.80 |
| 16 | 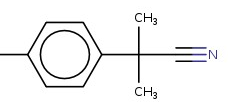 | 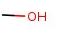 | -41.37 | -42.44 |
| 17 | 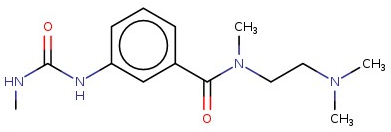 | 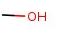 | -21.13 | -53.66 |
| **18** | **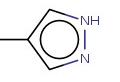** | **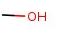** | **-45.12** | **-46.96** |
| **19** | **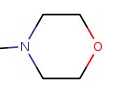** | **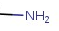** | **-38.32** | **-43.70** |
| 20 | 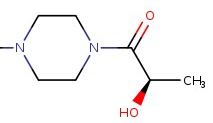 | 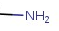 | -29.33 | -47.98 |
| 21 | 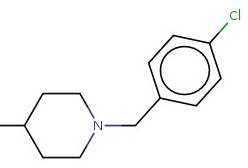 | 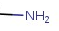 | -22.65 | -35.85 |
| 22 | 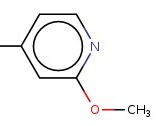 | 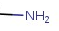 | -47.94 | -43.55 |
| 23 | 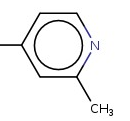 | 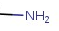 | -33.66 | -43.41 |
| 24 | 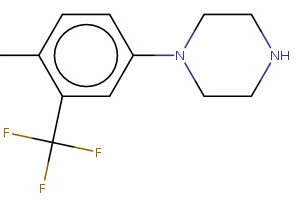 | 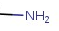 | 13.52 | -43.63 |
| 25 | 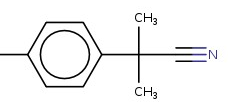 | 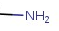 | -37.03 | -43.03 |
| 26 | 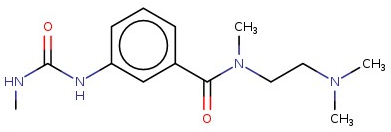 | 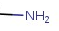 | 23.40 | -33.19 |
| 27 | 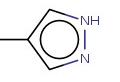 | 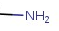 | -29.54 | -41.97 |
| **28** | **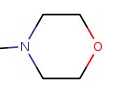** | **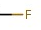** | **-48.59** | **-48.32** |
| 29 | 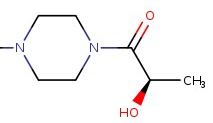 | 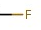 | -30.24 | -42.02 |
| 30 | 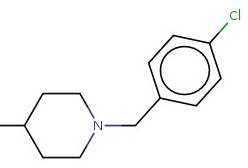 | 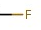 | -14.94 | -45.99 |
| 31 | 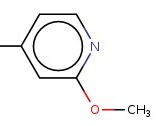 | 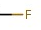 | -39.93 | -37.04 |
| 32 | 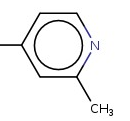 | 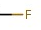 | -30.68 | -36.94 |
| 33 | 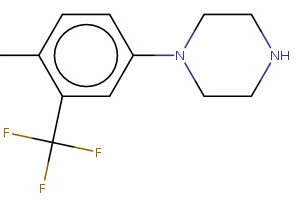 | 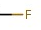 | -31.56 | -43.80 |
| 34 | 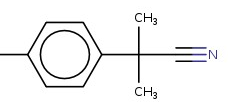 | 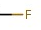 | -36.19 | -43.59 |
| 35 | 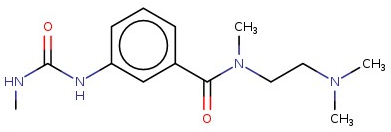 | 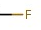 | -46.52 | -44.37 |
| 36 | 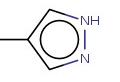 | 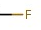 | -32.44 | -43.63 |
| 37 | 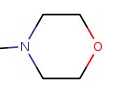 | 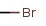 | -25.80 | -27.27 |
| **38** | **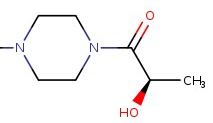** | **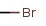** | **-42.95** | **-44.91** |
| 39 | 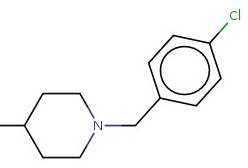 | 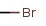 | -7.08 | -32.08 |
| 40 | 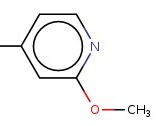 | 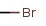 | -29.84 | -44.29 |
| 41 | 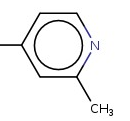 | 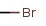 | -33.89 | -41.23 |
| 42 | 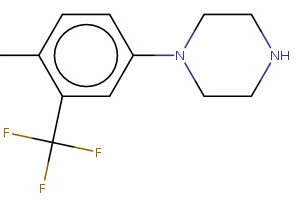 | 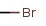 | -24.28 | -41.64 |
| 43 | 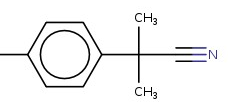 | 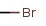 | -36.49 | -38.13 |
| 44 | 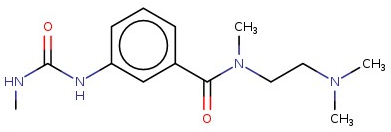 | 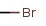 | -27.53 | -38.44 |
| 45 | 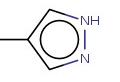 | 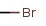 | -40.31 | -43.37 |
