# Supplementary material for: Anticancer compound XL765 as PI3K/mTOR dual inhibitor: A structural insight into the inhibitory mechanism using computational approaches
Source: PLoS One. 2019 Jun 27;14(6):e0219180. doi: 10.1371/journal.pone.0219180 (PMC6597235; doi:10.1371/journal.pone.0219180)
Supplement: S2 Table — (DOC) [file pone.0219180.s002.doc]

S2 Table. The human PI3Kγ residues interacting with compound 28 are listed with the number of hydrogen bonds, number of non-bonding interactions, and ΔASA.

| **Residues** | **Hydrogen bonds** | **Non-bonding interactions** | **ΔASA (Å^2^)** |
| --- | --- | --- | --- |
| Met-804 |  | 4 | 34.75 |
| Ser-806 |  | 1 | 33.08 |
| Trp-812 |  | 5 | 33.89 |
| Ile-831 |  | 2 | 24.91 |
| Ile-879 |  | 1 | 20.22 |
| Ala-885 |  | 3 | 19.85 |
| Thr-887 | 1 | 2 | 7.62 |
| Lys-890 | 1 | 3 | 41.55 |
| Asp-950 |  | 3 | 37.38 |
| Asn-951 |  | 2 | 15.86 |
| Met-953 |  | 4 | 35.54 |
| Ile-963 |  | 4 | 26.77 |
| Asp-964 |  | 3 | 35.62 |
