# Supplementary material for: Anticancer compound XL765 as PI3K/mTOR dual inhibitor: A structural insight into the inhibitory mechanism using computational approaches
Source: PLoS One. 2019 Jun 27;14(6):e0219180. doi: 10.1371/journal.pone.0219180 (PMC6597235; doi:10.1371/journal.pone.0219180)
Supplement: S4 Table — (DOC) [file pone.0219180.s004.doc]

S4 Table. The human PI3Kγ residues interacting with compound 38 are listed with the number of hydrogen bonds, number of non-bonding interactions, and ΔASA.

| **Residues** | **Hydrogen bonds** | **Non-bonding interactions** | **ΔASA (Å^2^)** |
| --- | --- | --- | --- |
| Met-804 |  | 8 | 43.6 |
| Ser-806 |  | 4 | 40.08 |
| Lys-807 | 1 | 1 | 46.77 |
| Trp-812 |  | 1 | 28.3 |
| Ile-879 |  | 1 | 24.92 |
| Ala-885 |  | 1 | 12.96 |
| Lys-890 | 2 | 3 | 46.21 |
| Asp-950 |  | 2 | 35.76 |
| Met-953 |  | 3 | 32.92 |
| Ile-963 |  | 1 | 26.77 |
| Asp-964 |  | 4 | 46.34 |
| His-967 | 1 | 3 | 39.37 |
| Leu-1090 |  | 5 | 37.92 |
