# Supplementary material for: Anticancer compound XL765 as PI3K/mTOR dual inhibitor: A structural insight into the inhibitory mechanism using computational approaches
Source: PLoS One. 2019 Jun 27;14(6):e0219180. doi: 10.1371/journal.pone.0219180 (PMC6597235; doi:10.1371/journal.pone.0219180)
Supplement: S5 Table — (DOC) [file pone.0219180.s005.doc]

S5 Table. The human PI3Kγ residues interacting with compound 9 are listed with the number of hydrogen bonds, number of non-bonding interactions, and ΔASA.

| **Residues** | **Hydrogen bonds** | **Non-bonding interactions** | **ΔASA (Å^2^)** |
| --- | --- | --- | --- |
| Ser-806 |  | 6 | 40.58 |
| Lys-807 |  | 12 | 76.04 |
| Ile-831 |  | 1 | 13.06 |
| Lys-833 |  | 2 | 25.98 |
| Asp-964 |  | 4 | 42.48 |
| His-967 |  | 3 | 53.79 |
| His-1089 |  | 2 | 25.47 |
| Leu-1090 |  | 6 | 46.22 |
| Val-1091 |  | 1 | 22.74 |
| Leu-1092 |  | 1 | 11.52 |
