# Supplementary material for: Anticancer compound XL765 as PI3K/mTOR dual inhibitor: A structural insight into the inhibitory mechanism using computational approaches
Source: PLoS One. 2019 Jun 27;14(6):e0219180. doi: 10.1371/journal.pone.0219180 (PMC6597235; doi:10.1371/journal.pone.0219180)
Supplement: S6 Table — (DOC) [file pone.0219180.s006.doc]

S6 Table. The human PI3Kγ residues interacting with compound 10 are listed with the number of hydrogen bonds, number of non-bonding interactions, and ΔASA.

| **Residues** | **Hydrogen bonds** | **Non-bonding interactions** | **ΔASA (Å^2^)** |
| --- | --- | --- | --- |
| Thr-887 | 1 |  | 7.55 |
| Met-804 |  | 1 | 37.55 |
| Ala-805 |  | 2 | 43 |
| Ser-806 |  | 2 | 36.08 |
| Lys-833 |  | 2 | 18.73 |
| Tyr-867 |  | 4 | 8.08 |
| Ile-879 |  | 3 | 20.52 |
| Lys-890 | 1 | 11 | 47.69 |
| Asp-950 |  | 6 | 34.24 |
| Met-953 |  | 1 | 22.73 |
| Ile-963 |  | 9 | 26.77 |
| Asp-964 |  | 11 | 47.03 |
