# Supplementary material for: Anticancer compound XL765 as PI3K/mTOR dual inhibitor: A structural insight into the inhibitory mechanism using computational approaches
Source: PLoS One. 2019 Jun 27;14(6):e0219180. doi: 10.1371/journal.pone.0219180 (PMC6597235; doi:10.1371/journal.pone.0219180)
Supplement: S7 Table — (DOC) [file pone.0219180.s007.doc]

S7 Table. The human PI3Kγ residues interacting with compound 19 are listed with the number of hydrogen bonds, number of non-bonding interactions, and ΔASA.

| **Residues** | **Hydrogen bonds** | **Non-bonding interactions** | **ΔASA (Å^2^)** |
| --- | --- | --- | --- |
| Val-1091 | 1 | 0 | 30.75 |
| Ala-805 |  | 1 | 60.48 |
| Ser-806 |  | 5 | 37.81 |
| Lys-890 |  | 3 | 45.82 |
| Asp-950 |  | 4 | 47.45 |
| Asn-951 |  | 2 | 15.2 |
| Asp-964 |  | 2 | 20.04 |
| His-967 |  | 5 | 32.18 |
| Leu-1090 | 1 | 2 | 31.2 |
