# Supplementary material for: Anticancer compound XL765 as PI3K/mTOR dual inhibitor: A structural insight into the inhibitory mechanism using computational approaches
Source: PLoS One. 2019 Jun 27;14(6):e0219180. doi: 10.1371/journal.pone.0219180 (PMC6597235; doi:10.1371/journal.pone.0219180)
Supplement: S8 Table — (DOC) [file pone.0219180.s008.doc]

S8 Table. The human mTOR residues interacting with compound 28 are listed with the number of hydrogen bonds, number of non-bonding interactions, and ΔASA.

| **Residues** | **Hydrogen bonds** | **Non-bonding interactions** | **ΔASA (Å^2^)** |
| --- | --- | --- | --- |
| Ser-2165 |  | 1 | 19.28 |
| Gln-2167 |  | 1 | 26.05 |
| Leu-2185 |  | 5 | 30.95 |
| Glu-2190 |  | 2 | 23.99 |
| Ile-2237 |  | 1 | 27.81 |
| Gly-2238 |  | 1 | 3.92 |
| Trp-2239 |  | 2 | 30.88 |
| Val-2240 |  | 2 | 8.93 |
| Met-2345 |  | 1 | 26.91 |
| Ile-2356 |  | 7 | 44.74 |
| Asp-2357 |  | 8 | 46.01 |
