# Supplementary material for: Anticancer compound XL765 as PI3K/mTOR dual inhibitor: A structural insight into the inhibitory mechanism using computational approaches
Source: PLoS One. 2019 Jun 27;14(6):e0219180. doi: 10.1371/journal.pone.0219180 (PMC6597235; doi:10.1371/journal.pone.0219180)
Supplement: S9 Table — (DOC) [file pone.0219180.s009.doc]

S9 Table. The human mTOR residues interacting with compound 18 are listed with the number of hydrogen bonds, number of non-bonding interactions, and ΔASA.

| **Residues** | **Hydrogen bonds** | **Non-bonding interactions** | **ΔASA (Å^2^)** |
| --- | --- | --- | --- |
| Ile-2163 |  | 1 | 20.47 |
| Leu-2185 |  | 3 | 30.4 |
| Tyr-2225 |  | 4 | 3.33 |
| Ile-2237 |  | 1 | 10.83 |
| Gly-2238 |  | 1 | 3.92 |
| Trp-2239 |  | 5 | 48.07 |
| Val-2240 |  | 2 | 8.46 |
| Cys-2243 |  | 1 | 13.36 |
| Asp-2244 |  | 1 | 11.21 |
| Thr-2245 | 1 | 9 | 30.4 |
| His-2247 |  | 2 | 25.17 |
| Ala-2248 |  | 1 | 12.99 |
| Arg-2251 | 1 | 1 | 24.69 |
| Ser-2342 |  | 3 | 28.36 |
| Ile-2356 |  | 4 | 35.08 |
