# Supplementary material for: Anticancer compound XL765 as PI3K/mTOR dual inhibitor: A structural insight into the inhibitory mechanism using computational approaches
Source: PLoS One. 2019 Jun 27;14(6):e0219180. doi: 10.1371/journal.pone.0219180 (PMC6597235; doi:10.1371/journal.pone.0219180)
Supplement: S10 Table — (DOC) [file pone.0219180.s010.doc]

S10 Table. The human mTOR residues interacting with compound 38 are listed with the number of hydrogen bonds, number of non-bonding interactions, and ΔASA.

| **Residues** | **Hydrogen bonds** | **Non-bonding interactions** | **ΔASA (Å^2^)** |
| --- | --- | --- | --- |
| Ser-2165 |  | 6 | 32.66 |
| Lys-2166 |  | 3 | 33.23 |
| Gln-2167 |  | 1 | 17.53 |
| Pro-2169 |  | 1 | 11.44 |
| Leu-2185 |  | 3 | 30.05 |
| Lys-2187 |  | 1 | 12.06 |
| Trp-2239 |  | 7 | 44.09 |
| Thr-2245 |  | 3 | 26.56 |
| Met-2345 |  | 3 | 29.73 |
| Ile-2356 |  | 3 | 39.04 |
