# Supplementary material for: Anticancer compound XL765 as PI3K/mTOR dual inhibitor: A structural insight into the inhibitory mechanism using computational approaches
Source: PLoS One. 2019 Jun 27;14(6):e0219180. doi: 10.1371/journal.pone.0219180 (PMC6597235; doi:10.1371/journal.pone.0219180)
Supplement: S12 Table — (DOC) [file pone.0219180.s012.doc]

S12 Table. The human mTOR residues interacting with compound 10 are listed with the number of hydrogen bonds, number of non-bonding interactions, and ΔASA.

| **Residues** | **Hydrogen bonds** | **Non-bonding interactions** | **ΔASA (Å^2^)** |
| --- | --- | --- | --- |
| Val-2240 | 2 | 0 | 8.98 |
| Ile-2163 |  | 1 | 35.98 |
| Pro-2169 |  | 1 | 11.04 |
| Leu-2185 |  | 1 | 28.84 |
| Tyr-2225 |  | 5 | 3.59 |
| Ile-2237 |  | 1 | 11.22 |
| Gly-2238 |  | 1 | 3.92 |
| Trp-2239 |  | 6 | 46.27 |
| His-2247 |  | 2 | 19.63 |
| Ser-2342 |  | 1 | 26.73 |
| Met-2345 |  | 3 | 30.25 |
| Ile-2356 |  | 4 | 38.62 |
