# Supplementary material for: Anticancer compound XL765 as PI3K/mTOR dual inhibitor: A structural insight into the inhibitory mechanism using computational approaches
Source: PLoS One. 2019 Jun 27;14(6):e0219180. doi: 10.1371/journal.pone.0219180 (PMC6597235; doi:10.1371/journal.pone.0219180)
Supplement: S13 Table — (DOC) [file pone.0219180.s013.doc]

S13 Table. The human mTOR residues interacting with compound 19 are listed with the number of hydrogen bonds, number of non-bonding interactions, and ΔASA.

| **Residues** | **Hydrogen bonds** | **Non-bonding interactions** | **ΔASA (Å^2^)** |
| --- | --- | --- | --- |
| Gly-2238 | 1 | 0 | 3.92 |
| Ile-2163 |  | 1 | 36.8 |
| Pro-2169 |  | 1 | 11.33 |
| Leu-2185 |  | 3 | 31.15 |
| Lys-2187 |  | 2 | 15.22 |
| Glu-2190 |  | 3 | 17.74 |
| Leu-2192 |  | 1 | 7.07 |
| Asp-2195 |  | 2 | 10.46 |
| Tyr-2225 |  | 2 | 7.69 |
| Ile-2237 |  | 4 | 27.85 |
| Trp-2239 |  | 6 | 49.03 |
| Met-2345 |  | 3 | 30.06 |
| Ile-2356 |  | 5 | 44.74 |
| Asp-2357 |  | 9 | 30.28 |
